# Impact of Dark Polariton States on Collective Strong Light–Matter Coupling in Molecules

**DOI:** 10.1021/acs.jpclett.5c01480

**Published:** 2025-07-25

**Authors:** Lucas Borges, Thomas Schnappinger, Markus Kowalewski

**Affiliations:** Department of Physics, 132068 Stockholm University, AlbaNova University Center, SE-106 91 Stockholm, Sweden

## Abstract

Polaritonic chemistry investigates the possible modification of chemical and photochemical reactions by means of strong light–matter coupling in optical cavities, as demonstrated in numerous experiments over the past few years. These experiments are typically interpreted in terms of the Jaynes–Cummings or Tavis–Cummings models under the assumption that the molecular ensemble is only excited by a single photon. In such a model, two polariton states compete with an overwhelming number of dark states, inhibiting polaritonic reactions entropically. We analyze the higher excitation manifolds of the Tavis–Cummings model along with a three-level system that resembles photochemical reactions. We demonstrate that allowing for more than a single excitation makes the reaction of the involved polaritons entropically more favorable.

## Graphical abstract

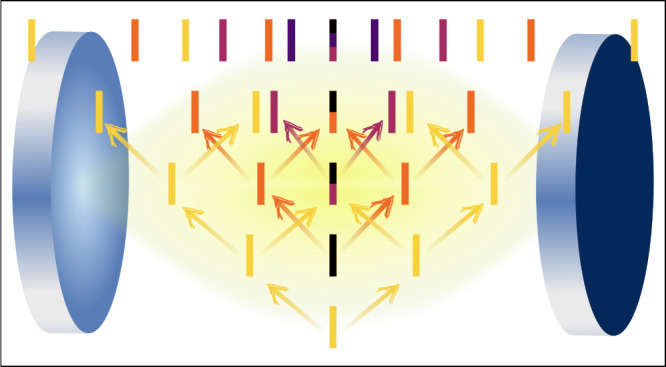

Polaritons are hybrid light–matter states that can form when matter transitions are strongly coupled to the electromagnetic field in an optical cavity.^1–6^ These states occur when the light–matter interaction rate is faster than the individual photon and matter decay rates in the system. Molecular polaritons have become an emerging area of research at the interface of quantum optics, chemistry, and materials science.^7–14^ Exciton polaritons, which combine electronic excitations with confined light modes, can alter the properties of excitonic systems, influencing processes such as electron transport,^15–21^ light harvesting,^22,23^ or energy transfer.^24–26^ When confined light modes are coupled to molecular vibrations, changes in molecular properties^27^ and chemical reactivity^28,29^ have been reported from experiments. Modified and extended versions of the Rabi and Dicke model^30,31^ or the Jaynes–Cummings and Tavis–Cummings (TC) model^32,33^ are often used to interpret the formation of hybrid polaritonic states and the outcome of polaritonic experiments. Despite considerable efforts, the fundamental theoretical understanding of these experiments is still incomplete.

Beyond these quantum-optical models, more recently ab initio methods such as quantum electrodynamics density functional theory^34^ and coupled cluster theory^35^ have been developed. However, in contrast to these methods, TC models offer a particularly efficient way to simulate large numbers of molecules.^18,36–40^ As shown in the literature,^41–45^ TC models can also be extended to include aspects such as nuclear degrees of freedom and permanent dipole moments relevant for molecular systems. Another advantage of models based on the TC Hamiltonian is that they allow a straightforward separation of the states of the coupled system into manifolds with an equal number of excitations in the molecular and photonic parts. Much of the research applying these models has focused on the first excitation manifold, where the formation of two polaritonic states the lower polariton (LP) state and the (UP) state, as well as dark states, in the case of more than one molecule is well understood. These dark states are decoupled from the cavity field in a pure Hamiltonian model due to symmetry, but become weakly coupled in the presence of dephasing or disorder. Due to their large number, they play an important role in a statistical mechanics picture, especially in open quantum systems.^41–43,46–48^ In the probably more realistic scenario, which allows for more excitations in the coupled system, additional types of hybrid states arise.^49–51^ The excitation of polaritons and dark states associated with lower excitation manifolds gives rise to different types of hybrid states, called multi polaritons and dark polaritons in the literature.^49,50^ These new additional hybrid states lead to more complex eigenvalue patterns in the higher excitation manifolds.

In this work, we investigate the influence of the eigenvalue structure of higher excitation manifolds of the TC model on reactions under the influence of electronic strong coupling. We extend the TC model to a three-level system that allows us to model photochemical reactions, such as singlet fission or triplet–triplet annihilation, which can be influenced by strong light–matter coupling.^15–20,52,53^ The letter is structured as follows. In the first part, the Hamiltonian for the standard TC model and extended TC model with an additional excited molecular state is introduced. To set the stage, we discuss the eigenvalue structure of the TC Hamiltonian and its extended version for higher excitation manifolds. In the final part, we present dissipative dynamics for the model systems and discuss the mechanisms and difference with the first excitation manifold.

All simulations in this study are based on the TC Hamiltonian^33^ for *N* identical two-level molecules or three-level molecules. In the case of two-level systems, we assume that the electronic states of a molecule can be described by two states |*g*⟩ and |*e*⟩, which are coupled to a single mode of an optical cavity. In the following, we use atomic units (ℏ = 4*π ε*_0_ = *m*_*e*_ = 1). The TC Hamiltonian reads:

$${Ĥ}_{TC}=\omega_{eg}\overset{N}{\underset{i=1}{\sum }}\left|e_{i}\right\rangle \left\langle e_{i}\right|+\omega_{c}{â}^{†}â+g_{c}\left({â}^{†}{Ŝ}^{-}+â{Ŝ}^{+}\right)$$ 1

where ω_*eg*_ = ω_*e*_ – ω_*g*_ is the energy difference between the two molecular energy levels |*e*⟩ and |*g*⟩, and ω_*c*_ is the frequency of the cavity mode. Here, *â* ^†^ and *â* are the bosonic creation and annihilation operators for the cavity mode. The coupling of the molecular excitation and the photon mode in the dipole approximation^54^ is described by the cavity coupling strength $g_{c}=\mu_{\mathrm{ge}}\sqrt{4\pi \omega_{c}/V_{c}}$ , where *V*_*c*_ is the quantization volume of the cavity and μ_*ge*_ is the transition dipole moment between the states |*g*⟩ and |*e*⟩. The first term in eq 1 describes the molecular excitations, the second term describes the excitation of the cavity mode, and the third term describes the coupling of molecular excitations to the cavity mode under the assumption of the rotating wave approximation.^54^ The molecular excitation and de-excitation operators *Ŝ* ^±^ are given by

2 $${Ŝ}^{\pm }=\overset{N}{\underset{i=1}{\sum }}{\mathrm{\sigma ̂}}_{i}^{\pm }$$

3 $${Ŝ}_{z}=\overset{N}{\underset{i=1}{\sum }}\frac{1}{2}\left({\mathrm{\sigma ̂}}_{i}^{-}{\mathrm{\sigma ̂}}_{i}^{-}-{\mathrm{\sigma ̂}}_{i}^{+}{\mathrm{\sigma ̂}}_{i}^{+}\right)$$

where σ̂_*i*_^+^ = (σ̂_*i*_^–^)^†^ = |*e*_*i*_⟩⟨*g*_*i*_| is the local Pauli operator exciting the *i*-th molecule from state |*g*⟩ to |*e*⟩. The operator representing the total number of excitations is given by

4 $${\mathrm{N̂}}_{x,\mathrm{TC}}=\overset{N}{\underset{i=1}{\sum }}{\mathrm{\sigma ̂}}_{i}^{+}{\mathrm{\sigma ̂}}_{i}^{-}+{â}^{†}â$$

where the first term yields the total number of excited molecules, and the second term, *n̂* = *â* ^†^ *â*, yields the number of photons in the cavity mode. *N̂*_*x,TC*_ commutes with the Hamiltonian in eq 1, and its eigenstates can be grouped into sets of states that share the same number of excitations, which we refer to as excitation manifolds. Furthermore, we define the *Ŝ* ^2^ operator

5 $${Ŝ}^{2}={Ŝ}_{z}^{2}+\left({Ŝ}^{+}{Ŝ}^{-}+{Ŝ}^{-}{Ŝ}^{+}\right)$$

with eigenvalues given by

$${Ŝ}^{2}\left|\Psi \right\rangle =\frac{1}{2}S\left(S+1\right)\left|\Psi \right\rangle$$ 6

which will be used to classify groups of eigenstates and is a conserved quantity in the absence of dissipation.^31^ The cooperation number 2*S* = 0...*N* can be interpreted as the number of molecules that contribute to a collectively coupled state.

The eigenstates of the first excitation manifold with respect to eq 1, are the well-known LP and UP states and a set of *N* – 1 dark states for which the molecules are decoupled from the cavity mode. The observable effective Rabi-splitting scales with the number of particles actively coupled to the cavity mode, and for *N*_x,TC_ ≪ *N* and *N* ≫ 1 we can write

7 $$\Omega_{R}\approx \sqrt{8g_{c}^{2}S+{\left(\omega_{eg}-\omega_{c}\right)}^{2}}$$

Note that the effective splitting now depends on 2*S* which coincides with *N* for the maximally allowed value of *S*.

Next, we introduce a third molecular state |*t*⟩, which is coupled to the excited state |*e*⟩ through a constant coupling *c*_*et*_. Such a coupling may be caused, for example, by a nonadiabatic process or internal conversion between a singlet and a triplet state. We assume that |*t*⟩ is optically dark, i.e., it does not have a transition dipole moment with respect to states |*g*⟩ or |*e*⟩, and therefore does not couple with the cavity mode. Figure 1 shows the corresponding level diagrams for the uncoupled states and the partially diagonalized model with *N* > 1 and *N*_exc_ = 1 in the case of resonant coupling to a cavity. The bare energies of |*t*⟩ and |*e*⟩ are detuned in the given example, making the population transfer ineffective despite the coupling. However, the lower polariton state can be brought into resonance with |*t*⟩, making the hybridization of |*t*⟩ and |*e*⟩ more efficient.

**Figure 1:**
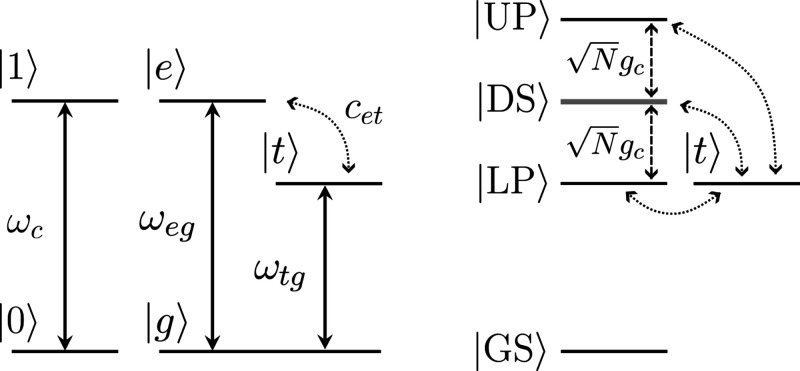
Three-level system scheme. Left panel: bare levels of the cavity and molecular systems for *N* = 1. Right panel: schematic representation of the partially diagonalized model with *N* > 1 and *N*_exc_ = 1. The cavity coupling between the excited state |*e*⟩ and the ground state |*g*⟩ leads to the formation of polariton states (LP and UP), as well as dark states (DS). The cavity coupling is chosen so that the |*t*⟩ state is approximately in resonance with the lower polariton state. This allows for a resonant population transfer between |*t*⟩ and LP, whereas the interaction with DS and UP is detuned.

The Hamiltonian from eq 1 is extended to a molecular three-level system, which then reads:

$$Ĥ={Ĥ}_{TC}+\omega_{tg}\overset{N}{\underset{i=1}{\sum }}\left|t_{i}\right\rangle \left\langle t_{i}\right|+c_{et}\left({Ŝ}_{t}^{+}+{Ŝ}_{t}^{-}\right)$$ 8

where ω_*tg*_ = ω_*t*_ – ω_*g*_ is the energy difference between states |*g*⟩ and |*t*⟩, and c_et_ describes the coupling between |*e*⟩ and |*t*⟩. Such a coupling may be caused, for example, by a non–adiabatic process or internal conversion between a singlet and a triplet state. We assume that |*t*⟩ is optically dark, i.e., it does not have a transition dipole moment between |*t*⟩ and |*e*⟩ or |*t*⟩ and |*g*⟩ and therefore does not couple to the cavity mode. The operators

9 $${Ŝ}_{t}^{+}=({Ŝ}_{t}^{-}{)}^{†}=\overset{N}{\underset{i=1}{\sum }}\left|e_{i}\right\rangle \left\langle t_{i}\right|$$

describe the transitions between |*t*⟩ and |*e*⟩ in the *i*th molecule. The number operator for the extended three-level system can then be written as *N̂*_*x*_ = *N̂*_x,TC_ + *N̂*_*t*_, where *N̂*_*t*_ = ∑_*i* = 1_^*N*^ |*t*_*i*_⟩⟨*t*_*i*_|. The expectation value ⟨*N̂*_*t*_⟩ quantifies the amount of |*t*⟩ excitation for a given state, or, in other words, its |*t*⟩ character. Since *N̂*_*x*_ commutes with Ĥ , *N̂*_*x*_ can be used to group the eigenstates by their excitation number. Both levels |*e*⟩ and |*t*⟩ are considered excited states, and thus the total number of states for a given *N*_*x*_ ≤ *N* is given by $\overset{N_{x}}{\underset{m=0}{\sum }}2^{m}\left(\begin{array}{c} N\\ m \end{array}\right)$ .

The uncoupled basis for TC Hamiltonian can be written as products of the bare molecular states |ϕ_*i*_⟩ ∈ |*g*_*i*_⟩,|*e*_*i*_⟩ and the Fock states |*n*⟩ of the cavity mode:

10 $$\left|\Psi \right\rangle =\left|\phi_{1}\right\rangle ⊗\cdot \cdot \cdot ⊗\left|\phi_{N}\right\rangle ⊗\left|n\right\rangle$$

The total number of states for a given number of excitations *N*_x,TC_ ≤ *N* is given by the sum of the binomial coefficients $\overset{N_{x}\mathrm{TC}}{\underset{i=0}{\sum }}\left(\begin{array}{c} N\\ i \end{array}\right)$ , corresponding to all combinations of *N*_x,TC_ excitations among the *N* molecules and the excitations of the cavity mode. In this study, we only take into account the cases where *N*_x,TC_ ≤ *N*. In the absence of counterrotating coupling terms, the ground state of the coupled system with *N*_x,TC_ = 0 is simply given by the product of all molecular ground states and the ground state of the cavity mode: |*G*,0⟩= ⊗_*i* = 1_^*N*^ |*g*_*i*_⟩⊗|*n* = 0⟩.

The Fabry-Pérot cavities that are typically used in polaritonic experiments^55^ have Q factors on the order of 100. Thus, photon decay from the cavity mode is an important process driving photochemical reactions described by the aforementioned three-level system. In addition, the dissipative channels that are opened by cavity decay and spontaneous emission from |*e*⟩ connect subspaces of different cooperation numbers *S* that are otherwise disconnected^43,56^ in a pure Schrödinger picture.

To describe the dynamics of the open-system and the density matrix ρ, we use a Lindblad master equation:

ρ̇(t)=−i[Ĥ(t)ρ(t)]+κ2(â†âρ(t)+ρ(t)â†â−2âρ(t)â†)+∑i=1NΓ2(ρ(t)|ei⟩⟨ei|+|ei⟩⟨ei|ρ(t)−2σ̂i−ρ(t)σ̂i+) 11

where κ is the photon decay rate and Γ is the spontaneous decay rate from |*e*⟩ to |*g*⟩. The corresponding Lindblad operators are thus *â* and σ̂_*i*_^–^, respectively. This choice of Lindblad operators is phenomenological and is adequate for the strong coupling regime.^57,58^

The TC Hamiltonian for two-level and three-level systems coupled to a single cavity mode, shown in eqs 1 and 8, was constructed and numerically diagonalized using the QuTiP package,^59^ version 5.0.2. The open system dynamics described by eq 11, is solved using the QuTiP package mesolve, which is based on the SciPy zvode ODE solver. All calculations were performed in a reproducible environment using the Nix package manager together with NixOS-QChem^60^ (commit 319ff67). We considered systems with *N* = 8 molecules and a maximum of *N*_*x*_ = 4 excitations located in the molecular subsystem. The initial state for the dynamics is constructed with *N*_*x*_ = 3 excitations located in the molecular subsystem. The open system dynamics is performed using adaptive time steps and the state populations are calculated every 5 fs. The decay rates for the dynamics are κ = 1/50 fs^–1^ and Γ = 1/1000 fs^–1^. In all static and dynamic simulations, the Hamiltonian is truncated at the *N*_*x*_-th excitation manifold. Within the rotating wave approximation this is a complete basis, since the subspaces with different excitation numbers are decoupled in the Hamiltonian. As the dissipative Lindblad dynamics only includes decay processes, higher excitation manifolds than the initial one are not accessible. To validate the use of the rotating wave approximation^54^ we performed simulations including the counter-rotating terms in the Hamiltonian of a system composed of *N* = 8 two-level emitters coupled to a cavity mode with *N*_*x*_ = 3 excitations. As counter rotating terms do not conserve energy, we truncated our basis to the excitation manifold of *N*_*x*_ = 5 to accommodate the possible increase in excitations. A direct comparison of the simulations with and without the rotating wave approximation is shown in Figures S8 and S9. To compare the dynamics of ensembles with different numbers of molecules, we maintain a constant collective coupling strength $g_{c}\sqrt{N}$ by scaling the single molecule coupling strength to ensure a consistent Rabi splitting for ensembles of different sizes. All parameters for the dynamics simulations are listed in Table S1 in the Supporting Information.

We now briefly discuss the eigenstates of the TC Hamiltonian, shown in eq 1, for excitation numbers beyond the well-studied case of one. The Hamiltonian can be divided into blocks of the same cooperation number *S*.^37,50^ These independent blocks are disconnected in the absence of dissipation and excitations of the molecules or the photon modes preserve *S*.

Figure 2(a) shows the eigenvalues of eq 1 for ω_*c*_ = ω_*eg*_ and *N* = 8. The vertical axis represents the excitation number (⟨*N̂*_x,TC_⟩) and the horizontal axis represents the relative shift with respect to the uncoupled eigenvalues λ. The inset numbers represent the number of degenerate states per eigenvalue, and the color code in (a) and (b) represents their normalized photon number (⟨*â* ^†^ *â*⟩/*N*_*x*_) and in (c) and (d) the cooperation number *S*. In Figures 2(b)–(d) the eigenstates are decomposed into different subgroups. The ground state and the dark states, which have no photonic character and do not experience any splitting, are shown in Figure 2(b). The dark states are only present for *N*_x,TC_ ≤ *N*/2, due to their highly asymmetric nature, but their number grows quickly with *N*_x,TC_.^61,62^

**Figure 2:**
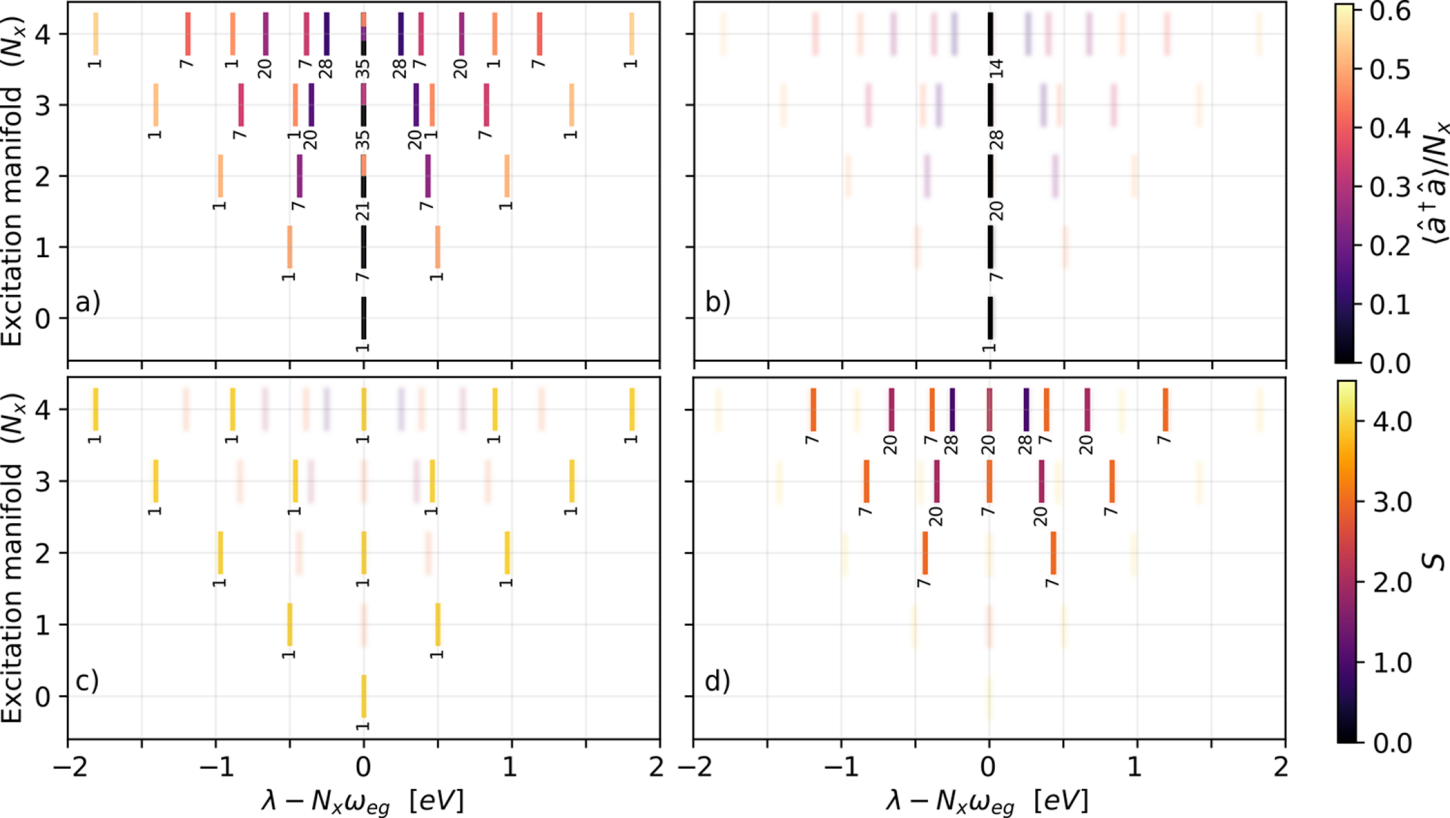
Energy diagrams for the eigenvalues (λ) of *N* = 8 two-level molecules resonantly coupled to a single cavity mode. The eigenenergies are shown as excitation number versus relative energy shift. The color code in panels (a) and (b) indicates the normalized photonic character (⟨*â* ^†^ *â*⟩/*N*_x,TC_) of each eigenstate group. We decompose these eigenstate groups into (b) dark states, (c) multi polaritons (*S* = *N*/2), and (d) dark polaritons (*S* < *N*/2 and ⟨*n̂*⟩ > 0). The inset numbers represent the number of degenerate states per eigenvalue. The color code in panels (c) and (d) indicates cooperation number *S*. The parameters are $g_{c}=0.5\;\mathrm{eV}/\sqrt{N}$ and ω_*eg*_ = ω_*c*_ = 4.3 eV.

Figure 2(c) shows the polariton states (*N*_*x*_ = 1) and the multi polariton states (*N*_*x*_ > 1). The polariton and multi polariton states are nondegenerate and their number scales with *N*_*x*_ + 1. The *N*_*x*_ excitations are distributed over all *N* molecules, resulting in a small overall effect/contribution for an individual molecule.^40,42,63,64^ Nevertheless, these states are typically considered for chemical reactivity under strong coupling. Transitions within this group of states are possible in a “diagonal” direction via *â* ^†^,*â* or *Ŝ* ^+^,*Ŝ* ^–^ and *ΔS* = 0.

Figure 2(d) shows the dark polaritons grouped by the cooperation numbers *S* < *N*/2. These states are created by further excitation of the dark states with *â* ^†^ or *Ŝ* ^+^. Their maximum energy splitting is smaller than the splitting of the multi polaritons^49,50,65–67^ (see eq 7). However, these states appear in degenerate groups, and thus provide a combinatorial advantage over multi polaritons which can be populated during a photochemical reaction. The ratio of dark polaritons to dark states for the dark polariton manifold generated by *N*_x,TC_ – 1 can be estimated for relative excitation numbers *c* = *N*_x,TC_/*N*:^61,62^

12 $$\frac{N_{DP}}{N_{D}}\approx \frac{N_{x}TC}{N-N_{x}TC}=\frac{c}{1-c}$$

For *N*_x,TC_ ≫ 1, this yields a significantly better ratio than for the nondegenerate multi polaritons. Figure 3(a) shows the ratios of dark polaritons to dark states in the dependence of the relative excitation numbers. The black line shows the particular ratio with the minimal possible *S*_*min*_ = *N*/2 – *N*_x,TC_ + 1 (i.e., the dark polaritons generated by exiting the dark states at *N*_x,TC_ – 1, see eq 12). Note that here at *c* = 0.1 the ratio is 1:10. Additional dark polaritons that have been generated from even lower lying dark state manifolds (blue and orange line) scale exponentially worse, and thus can be expected to have no significant contribution. In Figure 3(b) the dark polariton Rabi splitting relative to the multi polariton splitting is shown as a function of *c*. For *c* = 0.1 the Rabi splitting is still ≈ 90%. For a detailed derivation of the dark polariton ratios, see Supporting Information.

**Figure 3:**
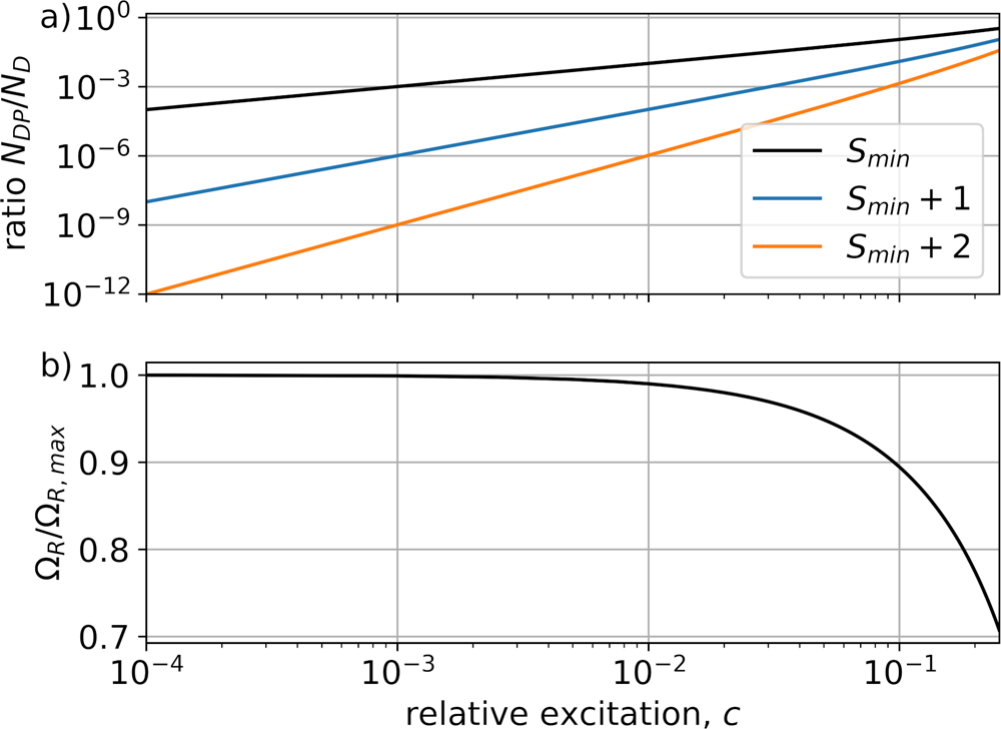
Scaling with respect to relative excitation. (a) Dark polariton to dark state ratios according to eq S6 for *S* = *N*/2 – *N*_x,TC_ + 1 (black), *S* = *N*/2 – *N*_x,TC_ + 2 (blue), and *S* = *N*/2 – *N*_x,TC_ + 3 (orange). (b) Dark polariton (*S*_*min*_) Rabi splitting relative to the multi polariton Rabi splitting, which scales with $\sqrt{1-2c}$ .

In the next step, we analyze the eigenvalues of the extended three-level model from eq 8. Figure 4 shows the eigenvalues color-coded by (a) photon number and (b) amount of *t* excitation defined by ⟨*N̂*_*t*_⟩. The number of possible states is much larger and some degeneracies in Figure 2 are lifted. However, some polariton group patterns, visible in Figure 2(a) can still be recognized. The collective cavity coupling, $\sqrt{N}g_{c}$ , is chosen so that |*t*⟩ is approximately resonant with the lower polariton branches, and thus mixing is mostly observed between |*t*⟩ and the lower polariton branches. The upper polariton branches are largely unaffected by coupling with |*t*⟩ since these are out of resonance with |*t*⟩ and thus exhibits little to no |*t*⟩ character.

**Figure 4:**
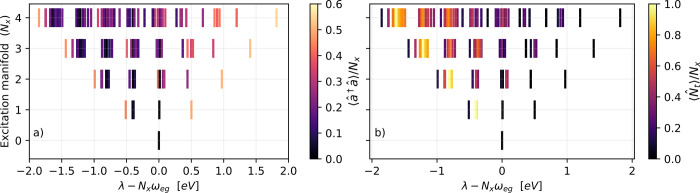
Energy diagrams for the eigenvalues (λ) of *N* = 8 three-level molecules resonantly coupled to a single cavity mode. The eigenenergies are shown as excitation number versus relative, cavity induced shift. The color code indicates the normalized expectation values of (a) ⟨*â* ^†^ *â*⟩/*N*_*x*_ and (b) ⟨*N̂*_*t*_⟩/*N*_*x*_ for each eigenstate. The parameters are $g_{c}=0.5\;\mathrm{eV}/\sqrt{N},\omega_{\mathrm{eg}}=\omega_{c}=4.3\;\mathrm{eV}$ , *c*_*et*_ = 0.05 eV and ω_*et*_ = 0.4 eV.

In the following, we compare the dynamics of the TC model with the two- and three-level systems in the presence of cavity and spontaneous decay. The dynamics is calculated according to the master eq eq 11. The decay of the cavity photons is modeled as a fast process with κ = 0.02 fs^–1^ and the spontaneous decay of |*e*⟩ as a slow process with Γ = 0.001 fs^–1^. The cavity coupling between |*g*⟩ and |*e*⟩ is considered to be in the collective strong coupling regime with $\sqrt{N}g_{c}=0.5\;\mathrm{eV}$ . For comparison, the dynamics of the two-level systems without a cavity is shown in Figure S5.

Figure 5(a) shows the time evolution of 8 two-level molecules, starting in a pure state with Tr­[ρ^2^] = 1 and three excitations in |*e*⟩. This is the most ideal case, which represents a superradiant state. The initial state density matrix has been constructed as ρ = |Φ⟩⟨Φ|, where |Φ⟩ = ∑_*i*_|ϕ_*i*_⟩ is the sum of all bare states |ϕ_*i*_⟩ with three excitations in |*e*⟩, and corresponds to a superposition of multi polariton states. The initial state (green) decays fast on the timescale of cavity decay (33 fs) and passes rapidly through the intermediate excitation manifolds *N*_*x*_ = 2 (orange) and *N*_*x*_ = 1 (blue). Note that the effective cavity decay rate is scaled by the photon number. At ≈ 600 fs nearly all population has reached the ground state (black line). Spontaneous decay creates a minor amount (10^–2^) of *N*_x,TC_ = 1 dark states that decay as expected with rate Γ.

**Figure 5:**
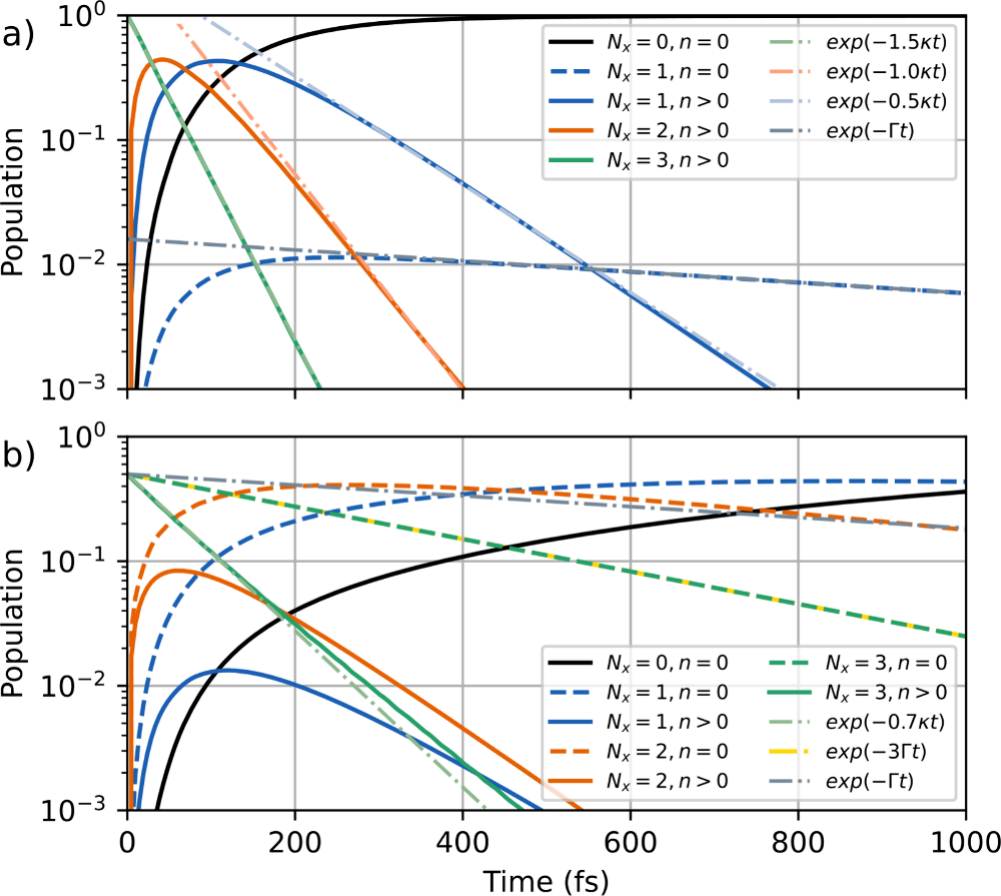
Population dynamics for *N* = 8 two-level molecules with (a) an initial state that represents a pure state with three molecular excitations *N*_*x*_ = 3 and is a superposition of four multi polariton states and (b) an initial state that represents a mixed stats. The populations are grouped by excitation number *N*_*x*_ and photonic character *n*. The parameters are $g_{c}=0.5\;\mathrm{eV}/\sqrt{N},\omega_{\mathrm{eg}}=\omega_{c}=4.3\;\mathrm{eV}$ , κ = 0.02 fs^–1^ and Γ = 0.001 fs^–1^.

Figure 5(b) shows the time evolution of 8 two-level molecules with their populations grouped into bright states (*n* > 0) and dark states *n* = 0 (solid and dashed lines, respectively). The initial state is constructed similarly as for the pure state, but with minimal purity (Tr­[ρ^2^] < 1, ρ_*i*≠*j*_ = 0). Here, the density matrix of this mixed state is given by ρ = ∑_*i*_|ϕ_*i*_⟩⟨ϕ_*i*_|, where |ϕ_*i*_⟩ are all bare states with three excitations in |*e*⟩. This type of state could be expected when the molecular state |*e*⟩ is populated by an incoherent excitation process or under thermal conditions via a chemical process. Here, the initial state with *N*_x,TC_ = 3 is composed of 50% dark state character (green, dashed line) and 50% multi polariton and dark polariton character (green, solid line). The dark state contribution decays according to the spontaneous decay rate (*N*_x,TC_Γ, yellow dashed-dotted line). The initial dark polariton and multi polariton states decay almost with the cavity decay rate (0.7κ, green dashed-dotted line). Due to the contribution of different *S* to the initial states, a large part of the initial decay populates the dark states with *N*_x,TC_ = 2 (orange, dashed line) and further into *N*_x,TC_ = 1 (blue, dashed line). The states with *N*_x,TC_ = 2 and *N*_x,TC_ = 1 decay with scaled rates *N*_x,TC_Γ (dark states *n* = 0) or close to the cavity decay rate κ (dark polariton and multi polariton states *n* > 0).

Subsequently, we show the dynamics for a corresponding three-level system with eight molecules. All other parameters are identical to those of the two-level system. The |*t*⟩ state is 0.4 eV below |*e*⟩ and the coupling between |*t*⟩ and |*e*⟩ is *c*_*et*_ = 0.05 eV, which corresponds to a 83 fs timescale. It is chosen to be weak compared to ω_*eg*_ and to the cavity coupling so that the interaction only slightly modifies the electronic states involved. The initial states are now constructed of three excitations in |*t*⟩ instead of |*e*⟩.

Figure 6(a) shows the time evolution starting in a pure state with Tr­[ρ] = 1 and three excited molecules in |*t*⟩. This state represents an ideal case where all molecules that are in state |*t*⟩ have been prepared by a coherent process. The initial state (green, solid line) thus is composed purely of multi polaritons from the lower polariton branch with a varying amount of |*t*⟩ character. These states decay rapidly, but their decay rate is now limited by the coupling *c*_*et*_. The initial state decays almost exclusively into a mixture of polariton states with *N*_*x*_ = 2 (orange, solid line), which in turn decays ≈ 3 times slower than the initial state. The subsequent populated states with *N*_*x*_ = 1 also contain a small number of dark states (blue, dashed line), which are populated by spontaneous decay processes. After 1 ps ≈ 50% has reached the ground state. The overall observed decay rates are now slower than in the case of the two-level system but are accelerated by the cavity compared to a system without a cavity (see Figure S6). This acceleration can be explained by the two features: first, the cavity coupling is chosen such that |*t*⟩ is resonant with the lower polariton states. Second, the cavity decay efficiently drives the polariton states toward the ground state.

**Figure 6:**
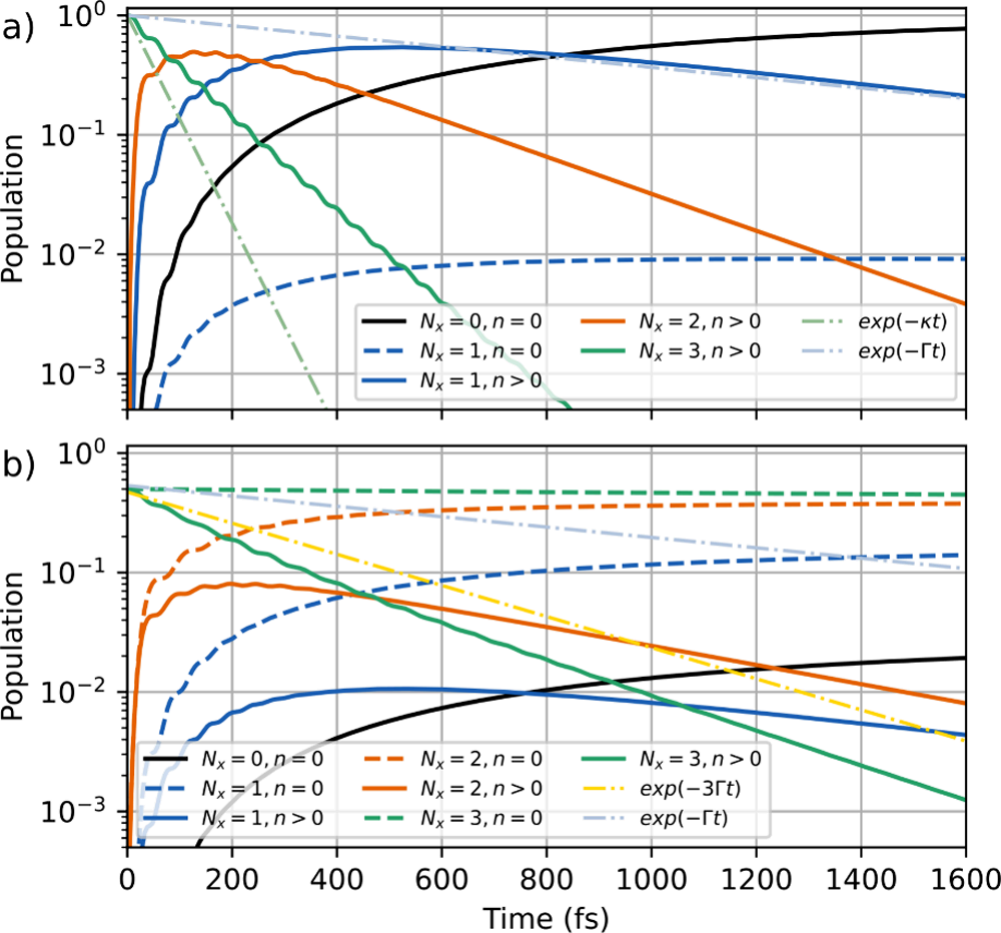
Population dynamics for *N* = 8 three-level molecules with an initial state that represents (a) a pure and (b) a mixed state with three molecular excitations in |*t*⟩. The population is grouped by the excitation number, and photonic character. The parameters are $g_{c}=0.5\;\mathrm{eV}/\sqrt{N},\omega_{\mathrm{eg}}=\omega_{c}=4.3\;\mathrm{eV}$ , *c*_*et*_ = 0.05 eV, ω_*et*_ = 0.4 eV, κ = 0.02 fs^–1^ and Γ = 0.001 fs^–1^.

Figure 6(b) shows the respective time evolution for the three-level system that starts in a mixed state. Here, 50% of the initial population is in dark states with a predominant |*t*⟩ character (green, dashed line), which shows an inhibited decay, since no direct decay channel for |*t*⟩ is included. The other 50% of the initial population are composed of a mixture of dark polaritons and multi polaritons, with a varying amount of |*t*⟩ character (green, solid line). This state decays faster than the spontaneous decay (yellow, dashed-dotted line). Note that parts of the dark polariton states now decay in the *N*_*x*_ = 2 dark states (orange, dashed line), where a large fraction of the population gets trapped. In contrast, the *N*_*x*_ = 2 polariton states (orange, solid line) decay further into *N*_*x*_ = 1 polariton and dark states (blue, solid and dashed lines, respectively). At 1 ps ≈ 98% of the population is located in dark states, but no significant amount has reached the global ground state. The overall decay of the |*t*⟩ state is slower than for the two-level system in Figure 5, but the decay is again accelerated by coupling to the cavity compared to a system without a cavity shown in Figure S6.

In Figures 5 and 6, we have shown a comparison of the ideal superradiant and a more realistic, incoherently prepared mixed state. The superradiant state is a perfect superposition and represents a pure state in terms of the density matrix. Such a state represents a linear combination of multi polariton eigenstates, or, in the case of the three-level system, a combination of multi polariton and dark polariton states. This state can reach the ground state very efficiently via cavity decay and is only limited by the cavity decay rate and, in the three-level model, additionally by the coupling between |*t*⟩ and |*e*⟩. Low photon numbers, which can be a result of quantum interference between upper and lower polariton states, can also lead to a delayed decay of the excitation. The mixed state, in contrast, could be expected for nonzero temperature conditions and represents an incoherent state preparation, and is therefore closest to realistic experimental conditions at room temperature. This state projects on all possible eigenstates, namely the multi polariton states, the dark polaritons, and the dark states. As can be seen in Figure 2, the number of degenerate states for each value of *S* becomes important. Here, 2*S* represents the number of molecules that are effectively coupled to the cavity mode. The multi polaritons with *S* = *N*/2 are not degenerate and thus provide the smallest number of states. These states suffer from the 1/*N* problem^40,63,64^ and have a negligible contribution to the initial state. For *N*_*x*_ = 1 there are *N* – 1 dark states that will dominate the superposition. However, for *N*_*x*_ > 1 dark polaritons are formed from dark states with *N*_*x*_ – 1, *N*_*x*_ – 2, ... and are present in a much larger number. In the discussed examples of two-level systems with *N*_x,TC_ = 3, the ratio of multi polariton and dark polariton states to dark states is approximately 2:1. This ratio improves with smaller cooperation numbers 2*S* (see eq 12), providing more states for hybridization and reactivity. However, dark polaritons introduce a reduced overall collective coupling strength, scaling with $\sqrt{2S}$ , because the excitations are distributed over fewer molecules. Note that the observed Rabi-splitting depends on *S*, but is otherwise nearly independent of the total number of excitations.

The generalization to large *N* (eq 12) shows that the relative number of excited molecules is decisive. For example, for *S* = *N*/2 – *N*_*x*_ + 1 (i.e., the dark polaritons generated by exciting *N*_*x*_ – 1) and a relative excitation number *c* = 0.01 the ratio of dark polariton states to dark states is 1:100, and thus there are sufficiently many bright states available for interaction with the cavity mode. The Rabi splitting in this scenario is still 99% of the maximum possible Rabi splitting, leaving the effective cavity coupling nearly unaffected. A partially excited sample may be advantageous for a chemical reaction in the presence of strong coupling. The collective Rabi splitting is not significantly affected, and the reaction can benefit from the energy shift of the polariton states. In contrast to exciting the system directly from the ground state, the ratio between dark polariton states and dark states presents an entropic advantage. Figure 6(b) illustrates this advantage: Even in the most unfavorable case, i.e., the incorrectly mixed state, the excited fraction can still perform at least one effective decay step. Dark polaritons cannot reach the global ground state because they decay to dark states with the same *S*, which limits the number of molecules that can undergo a cavity-assisted reaction. The overall decay is still more efficient than in the absence of cavity decay or in the absence of strong coupling.

It is important to note that linear spectroscopy alone may not be able to distinguish the state of the system in terms of the excitation number. Optical transitions are allowed only for *ΔN*_*x*_ = ± 1 and *ΔS* = 0. The observed Rabi-splitting scales with $\sqrt{2S/V_{c}}$ , and thus the density of molecules that are effectively coupled to the cavity modes. The total number of excitations *N*_*x*_ has no significant effect on the observed Rabi splitting, in the limit of large *N* and *N*_*x*_ ≪ *N*. More sophisticated spectroscopic schemes are thus needed to distinguish these additional degrees of freedom.

In summary, we have shown that states with higher excitation numbers in an ensemble of molecules resonantly coupled to a cavity provide a favorable density of states. The investigated three-level molecular model has broad applicability and shows that a photochemical reaction is still possible, despite the presence of a large number of dark states. Shifting the focus away from the (multi) polariton states, it is the group of dark polaritons that provides a large number of states to interact with, and thus gives rise to an entropic advantage. These states allow overcoming the 1/*N* problem in polaritonic chemistry which is typically cited as a paradox.^63^ The introduced model represents, for example, a triplet–triplet annihilation process,^52^ where a significant fraction of molecules is created in an excited state and the reaction is driven by photon decay. Our analysis also hints that in electronic strong coupling in general, only a fraction of the molecules in the cavity take part in a reaction. Moreover, higher excitation numbers may be needed to enable polaritonic reactions. It thus can be expected that the states that appear in the higher excitation manifolds are important for a statistical mechanics model that aims to describe reaction rates. To the best of our knowledge, the dark polariton states and their properties have not yet been studied in the context of molecular polaritons or dynamical processes in molecular ensembles. However, multi polaritonic states and dark polaritonic states are well studied in quantum optics to describe the effect of superradiance and the underlying many-body physical mechanism.^68–71^ The presented mechanism relies on such superradiance. For the mixed initial states, our results show that a single photon can interact efficiently with the dark polariton states and drive single-photon cycles. The important feature, however, is the ratio between the number of dark-polariton and dark states.

We have analyzed ensembles with a small number of molecules in the absence of energetic disorder and nuclear degrees of freedom. Taking into account these types of disorder present at finite temperature introduces mixing^39,43,72–74^ of polariton states and dark states and thus could be expected to further modify the density of states.

Our findings may have broader implications for the understanding of polaritonic chemistry as it sheds light on the underlying statistics and mechanism. The presented model fits well for electronic strong coupling, which involves electronically excited states. However, without loss of generality this model could be extended to investigate the statistics of vibrational strong coupled systems.

## Supplementary Material



## Data Availability

The data supporting this study’s findings are available from the corresponding author upon reasonable request.

## Notes

Detailed derivation of the ratio of dark states across excitation manifolds and additional plots of the evolution of the two- and three-level systems under strong light–matter coupling for different parameters and using the Dicke model (PDF)
