# Supplementary material for: Impact of Dark Polariton States on Collective Strong Light–Matter Coupling in Molecules
Source: J Phys Chem Lett. 2025 Jul 25;16(31):7807–15. doi: 10.1021/acs.jpclett.5c01480 (PMC12337150; doi:10.1021/acs.jpclett.5c01480)
Supplement: Supplementary file 1 [file jz5c01480_si_001.pdf]

Supporting Information:
The impact of dark polariton states for collective strong light-matter coupling in molecules

Lucas Borges, Thomas Schnappinger, and Markus Kowalewski*

Department of Physics, Stockholm University,

AlbaNova University Center, SE-106 91 Stockholm, Sweden

CONTENTS

S1. Scaling of dark state number in higher excitation manifolds 2

S2. Supplemental plots for the dynamics 4

References 13

* markus.kowalewski@fysik.su.se

S1. SCALING OF DARK STATE NUMBER IN HIGHER EXCITATION MANIFOLDS

A state $|\psi_{\mathrm{DS}}\rangle$ formed by N two-level molecules strongly coupled to a cavity mode is considered a dark state if it is an eigenstate of the Hamiltonian $\hat{\mathcal{H}}$ and satisfies $\langle\psi_{\mathrm{DS}}|\hat{a}^\dagger\hat{a}|\psi_{\mathrm{DS}}\rangle = 0$. For a manifold with $N_{\mathrm{x,TC}}$ excitations, the number of states that involve only excitations in the matter part and thus have zero photonic character is given by the binomial coefficient $\mathcal{M} = \binom{N}{N_{\mathrm{x,TC}}}$. Therefore, a dark state $|\psi_{\mathrm{DS}}\rangle$ within this manifold must be a linear combination of these $\mathcal{M}$ bare states, which can be expressed as:

$$|\psi_{\mathrm{DS}}\rangle = \sum_{i}^{\mathcal{M}} \alpha_i \hat{\mathcal{S}}_i^{N_{\mathrm{x,TC}}}|0\rangle, \tag{S1}$$

with

$$\hat{\mathcal{S}}_i^{N_{\mathrm{x,TC}}} = \bigotimes_{k\in\mathcal{C}_i} \hat{\sigma}_k^+, \tag{S2}$$

where $\mathcal{C}_i$ is the i-th set of the combinations of $\{1,\ldots,N\}$ choose $N_{\mathrm{x,TC}}$. The action of $\hat{\mathcal{S}}_i^{N_{\mathrm{x,TC}}}$ on the collective ground state $|0\rangle$ produces the i-th bare state, with α_i being its contribution to the dark state in Eq. (S1).

The action of the Hamiltonian on $|\psi_{\mathrm{DS}}\rangle$ is given by

$$\hat{\mathcal{H}}_{TC}|\psi_{\mathrm{DS}}\rangle = \sum_{k=1}^{N} \left(\omega_{eg}|e_k\rangle\langle e_k| + g_c\hat{a}^\dagger\hat{\sigma}_k^-\right)|\psi_{\mathrm{DS}}\rangle, \tag{S3}$$

$$\hat{\mathcal{H}}_{TC}|\psi_{\mathrm{DS}}\rangle = N_{\mathrm{x,TC}}\omega_{eg}|\psi_{\mathrm{DS}}\rangle + g_c\sum_{j=1}^{\mathcal{M}'} \beta_j \hat{\mathcal{S}}_j^{N_{\mathrm{x,TC}}-1}\hat{a}^\dagger|0\rangle, \tag{S4}$$

where $\mathcal{M}' = \binom{N}{N_{\mathrm{x,TC}}}$, and β_j are sums of the coefficients α_i such that $\hat{\mathcal{S}}_j^{N_{\mathrm{x,TC}}-1} = \hat{\sigma}_k^-\hat{\mathcal{S}}_i^{N_{\mathrm{x,TC}}}$, for any k.

For $|\psi_{\mathrm{DS}}\rangle$ to be an eigenstate of $\hat{\mathcal{H}}$, requires that $\beta_j = 0, \forall j$, which is equivalent to a homogeneous system of $\mathcal{M}'$ linear equations with $\mathcal{M}$ parameters. Infinite nontrivial solutions for this system exist only when $\mathcal{M} > \mathcal{M}'$ [1, 2]. The total number of dark states in the manifold is then the dimension of the non-trivial solutions for the homogeouns system of linear equations $\beta_j = 0$, which corresponds to the difference between $\mathcal{M}$ and $\mathcal{M}'$, and reads:

$$\mathcal{N}_{DS}(N, N_{\mathrm{x,TC}}) = \binom{N}{N_{\mathrm{x,TC}}} - \binom{N}{N_{\mathrm{x,TC}}-1} = \frac{N!(N-2N_{\mathrm{x,TC}}+1)}{N_{\mathrm{x,TC}}!(N-N_{\mathrm{x,TC}}+1)!}. \tag{S5}$$

Their cooperation number is given by $S = N/2 - N_{\mathrm{x,TC}}$. Consequently, we can deduce that there are no dark states for manifolds with $N_{\mathrm{x,TC}} > N/2 + 1$.

For the case of the second excitation manifold, the number of dark states is $\mathcal{N}_{DS}(N, 2) = N(N-3)/2$, while the number of dark polaritons is $2(N-1)$, arising from the splitting of the dark states present in the single-excitation manifold. The ratio of the lower dark polariton states to dark states can be estimated using Eq. S5 and the fact that dark polaritons are created by further exciting dark states. For dark polaritons with a given $N_{\mathrm{x,TC}}$ and a given quantum number S the ratio can be written as:

$$R_S = \frac{\mathcal{N}_{DS}(N, N_{\mathrm{x,TC}}/2 - S)}{\mathcal{N}_{DS}(N, N_{\mathrm{x,TC}})} = \frac{(2S+1)\prod_{p=N/2-S+1}^{N_{\mathrm{x,TC}}} p}{(N - 2N_{\mathrm{x,TC}} + 1)\prod_{p=N-N_{\mathrm{x,TC}}+2}^{N/2+S+1} p}\,. \tag{S6}$$

The minimum S for a dark polariton manifold with given $N_{\mathrm{x,TC}}$ is given by $S_i = N/2 - N_{\mathrm{x,TC}} + i$ with $i = 1$. In the limit of large N and $S \ll N/2$ and small i, Eq. S6 can approximated as

$$R_S = \frac{\mathcal{N}_{DS}(N, N_{\mathrm{x,TC}}/2 - S_i)}{\mathcal{N}_{DS}(N, N_{\mathrm{x,TC}})} \approx \frac{\prod_{p=N/2-S_i+1}^{N_{\mathrm{x,TC}}} p}{\prod_{p=N-N_{\mathrm{x,TC}}+2}^{N/2+S_i+1} p} \approx \left(\frac{N_{\mathrm{x,TC}}}{N - N_{\mathrm{x,TC}}}\right)^i = \left(\frac{c}{1-c}\right)^i, \tag{S7}$$

where we have replaced the excitation number with the relative excitation number $c = N_{\mathrm{x,TC}}/N$. The Rabi splitting relative to the maximum possible value is given by $\Omega_{rel} = \sqrt{2S_i/N}$, which for large N yields:

$$\Omega_{rel} \approx \sqrt{1 - \frac{2N_{\mathrm{x,TC}}}{N}} = \sqrt{1-2c}\,. \tag{S8}$$

Note, that for large N and small i, Ω_{rel} does not depend on i.

S2. SUPPLEMENTAL PLOTS FOR THE DYNAMICS

In this section, we provide additional plots for the dynamics of the two-level and three-level systems discussed in the manuscript. The parameters used for all dynamics simulations are listed in Table S1.

symbol	description	quantity
N	number of quantum emitters	8
g_c^N	collective coupling strength	0.5eV
ω_{eg}	energy transition between levels e and g	4.3eV
ω_{et}	energy transition between levels e and t	0.4eV
c_{et}	coherent coupling between levels e and t	0.05eV
κ	cavity decay rate	0.02fs^{-1}
Γ	quantum emitter spontaneous emission rate	0.001fs^{-1}
$N_x(0)$	initial state excitation number	3

TABLE S1. Parameters used to for the propagations of the two- and three level system models.

Fig. S1 shows the time evolution of eight two-level molecules on a linear scale, starting in the pure state with $\mathrm{Tr}\,[\rho^2] = 1$ and three excitations in $|e\rangle$. The populations are grouped by the number of excitations, the cooperation number, and the photonic character, showing that the dynamics involves mostly multi polaritons states with $S = N/2$. Only a small contribution from dark states is observed (blue dashed curve).

The time evolution of 8 two-level molecules is shown in Fig. S2 on a linear scale, starting in a mixed state with $\mathrm{Tr}\,[\rho^2] < 1$ and three excitations in $|e\rangle$. In Fig. S2(a) the populations are grouped by the excitation number $N_{\mathrm{x,TC}}$, cooperation number S and photonic character. The initial mixed state consists of dark states (50%), dark polaritons (35, 7% and 12, 5%), and multi polaritons (1, 8%). The states shown in Fig. S2(b) are grouped only by the excitation number $N_{\mathrm{x,TC}}$ and photonic character.

In Figs. S3 and S4 we show the dynamics of three-level systems with $N = 8$ and initially three excitations in $|t\rangle$ on a linear scale. The parameters used are identical to those of the two-level system. The $|t\rangle$ state is 0.4 eV below $|e\rangle$ and the coupling between $|t\rangle$ and $|e\rangle$ is $c_{et} = 0.05$ eV. The initial state for the dynamics shown in Fig. S3 has maximum purity,

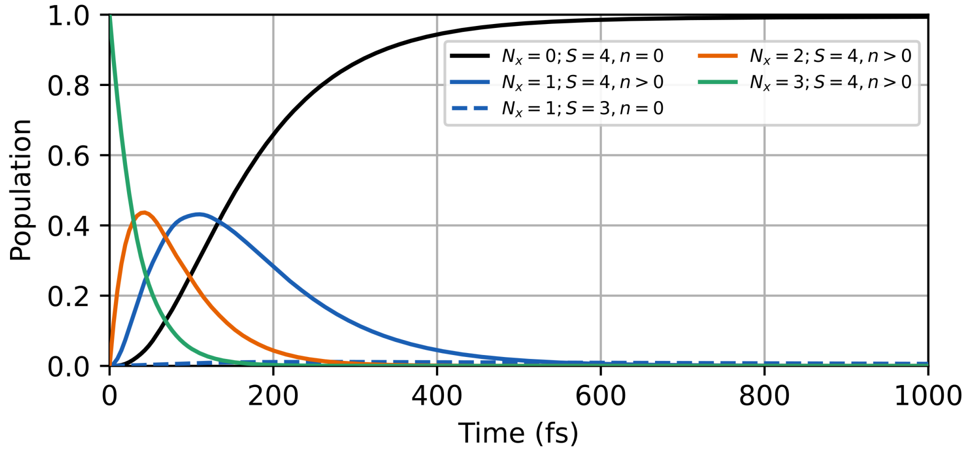

FIG. S1. Population dynamics for $N = 8$ two-level molecules with a initial state that represent a pure state with three molecular excitations and is a superposition of four multi polariton states with $N_{\mathrm{x,TC}} = 3$ and $S = 4$. The populations are grouped by the excitation number $N_{\mathrm{x,TC}}$, cooperation number S and photonic character. Only polariton states with $S = 4$ contribute to the populations. All parameters are shown in Table S1.

$\mathrm{Tr}\left[\rho^2\right] = 1$, and for Fig. S4 it has minimum purity. The expectation values of the operators $\hat{N}_t$, $\hat{N}_e$ and $\hat{n}$ are shown in Fig. S3(a) for the pure initial state and in Fig. S4(a) for the mixed initial state. In the case of a pure initial state, we observe significant oscillations between N_t and a combination of N_e and n, indicating an effective exchange between $|t\rangle$ and the cavity photon via $|e\rangle$. As a consequence, a sufficiently large number of photons allows an efficient decay channel through the cavity to the global ground state, leading to a fast decrease of all three expectation values over time. In contrast, the three expectation values behave differently for the mixed initial state. Within the first hundred femtoseconds, N_t decays three times slower than in the pure case, and for later times, the decay slows down even more. As a result, N_e and n remain very small, and a fast decay via the cavity to the global ground state is less efficient than in the case of a pure initial state. In Fig. S3(b) and Fig. S4(b), the state population is shown on a linear scale grouped by number of excitations and photonic character for both pure and mixed initial states.

As a reference for the two-level system and the three-level system with $N = 8$, we show in Fig. S5 and Fig. S6 the evolution of the system with initially three excitations outside a cavity by setting the cavity coupling to zero. Whether the initial state is pure, shown

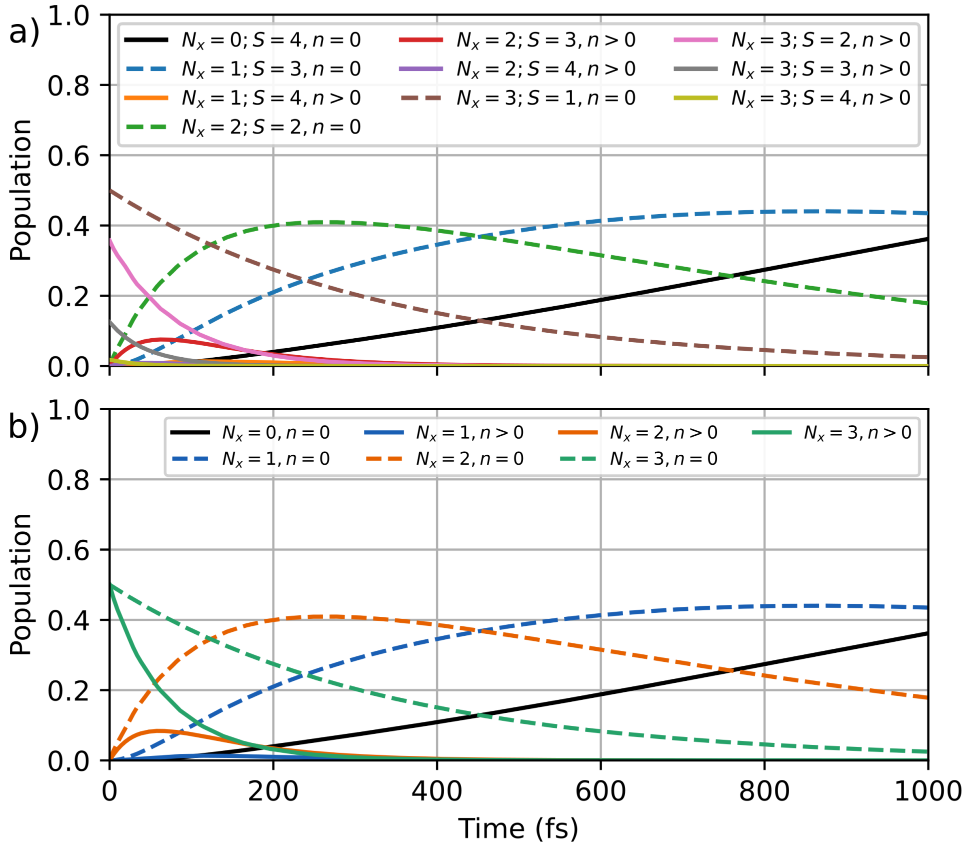

FIG. S2. Population dynamics for $N = 8$ two-level molecules with an initial state that represents a mixed state with three molecular excitations and is a superposition of multi-polaritons, dark polaritons, and dark states with $N_{\mathrm{x,TC}} = 3$. The populations are grouped in (a) by the excitation number $N_{\mathrm{x,TC}}$, cooperation number S and photonic character and in (b) only by $N_{\mathrm{x,TC}}$ and photonic character. All parameters are shown in Table S1.

in Fig. S5(a) and Fig. S6(a), or a mixed state, shown in Fig. S5(b) and Fig. S6(b), the observed population dynamics are very slow and nearly identical. The overall decay to the ground state is faster in the two-level case than in the three-level case. In the latter case, the only way to reach lower excitation manifolds is through spontaneous decay from the $|e\rangle$ state. This state is slowly populated becaause $|e\rangle$ and $|t\rangle$ are detuned without coupling to the cavity.

Additionally, we show the dynamics of the three-level system with $N = 8$ resonantly coupled to a cavity mode and starting in a mixed state with three excitations in $|t\rangle$ in Fig. S7 for different c_{et} couplings. All other parameters are identical to Fig. S7. For smaller

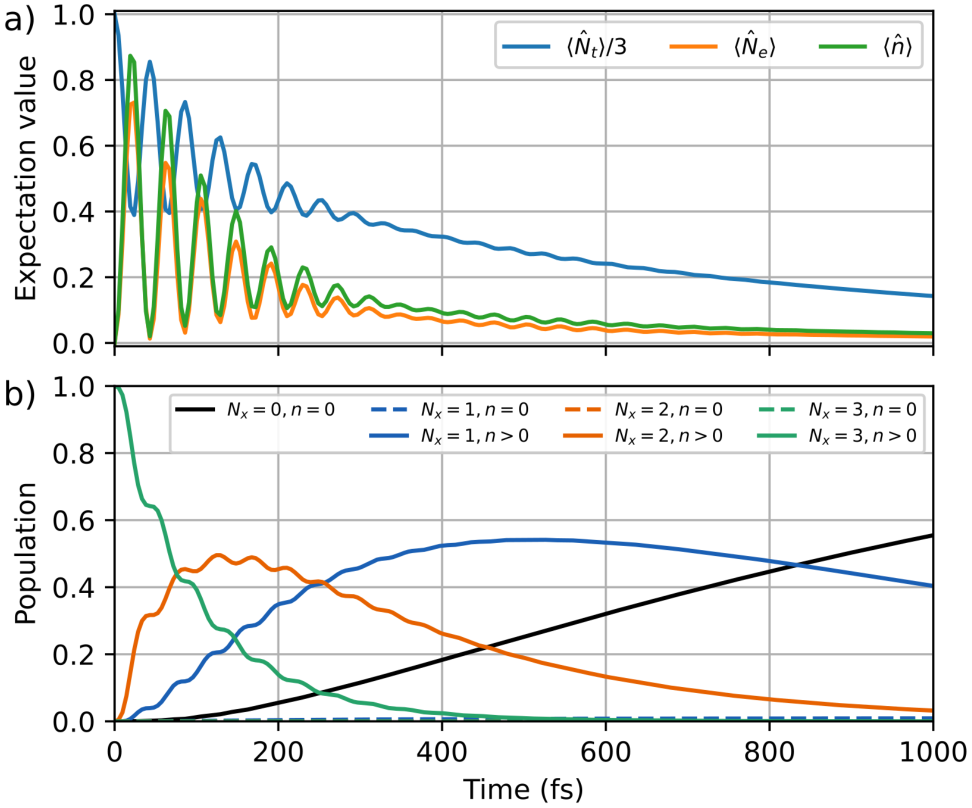

FIG. S3. Dynamics of a three-level system with $N = 8$ and an initial state that represents a pure state with three excitations in $|t\rangle$. (a) Expectation values of $\hat{N}_t$, $\hat{N}_e$ and $\hat{n}$, giving the quantity of t, e and photonic excitations in the system. (b) Population grouped by the excitation number and photonic character. All parameters are shown in Table S1.

coupling, $c_{et} = 0.025\,\text{eV}$ (half the initial value), shown in Fig. S7(a), the overall decay slows down significantly, while for large coupling, $c_{et} = 0.1\,\text{eV}$ (double the initial value), shown in Fig. S7(b), the decay in the global ground state increases.

In Figures S8 and S9, we compare the open system dynamics of the Tavis-Cummings model and the Dicke model for a system composed of $N = 8$ two-level emitters coupled to a cavity mode, with an initial state either pure or mixed, with $N_x = 3$ excitations. The Hamiltonian of the Dicke model is given by:

$$\hat{\mathcal{H}}_{\text{Dicke}} = \omega_{eg} \sum_{i=1}^{N} |e_i\rangle\langle e_i| + \omega_c \hat{a}^\dagger \hat{a} + g_c \left(\hat{a}^\dagger + \hat{a}\right) \left(\hat{S}^- + \hat{S}^+\right), \tag{S9}$$

including counter-rotating terms. Note that the dipole self-energy [3–5] is not included in the modeling. In Fig. S8 the same coupling strength of $g_c^N = 0.5$ eV is used as in the main

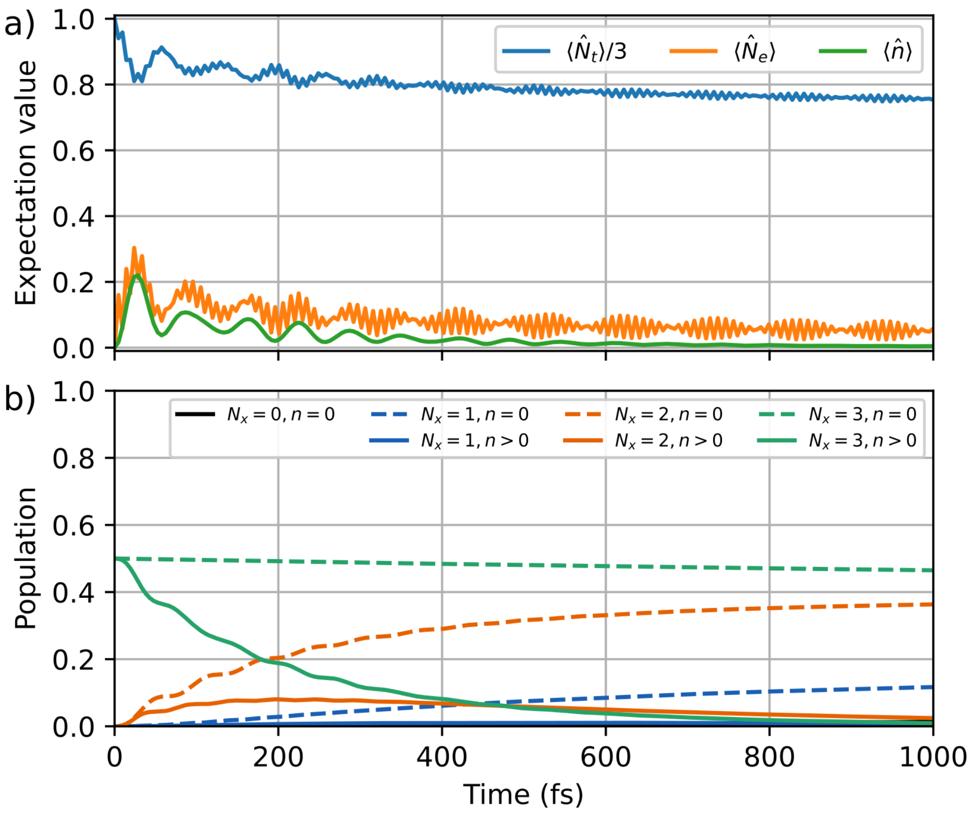

FIG. S4. Dynamics of a three-level system with $N = 8$ and an initial state that represents a mixed state with three excitations in $|t\rangle$. (a) Expectation values of $\hat{N}_t$, $\hat{N}_e$ and $\hat{n}$, giving the quantity of t, e and photonic excitations in the system. (b) Population grouped by the excitation number and photonic character. All parameters are shown in Table S1.

paper. The differences between the models are minimal, becoming noticeable after 200fs for the case with a pure initial state Fig.S8(a) and after 1500fs for the mixed initial state case Fig.S8(b). The system reaches a stationary state in the case of initial pure state, although the values are very small, as we can observe in the population difference for $N_x = 0$, which does not exceed the value of 10^{-2}. The populations of higher excitation manifolds ($N_x > 3$), which are only populated due to the contributions of counter-rotating terms, also do not exceed 10^{-2} and decay to values below 10^{-5}.

At a coupling strength of $g_c^N = 0.2\omega_c = 0.86$eV, the population differences for some manifolds exceed 10^{-2}, making the deviations noticeable at earlier times and also the population of higher excitation manifolds increases. However, the decay rates during the early time period remain only slightly perturbed, which does not affect the conclusions drawn in the

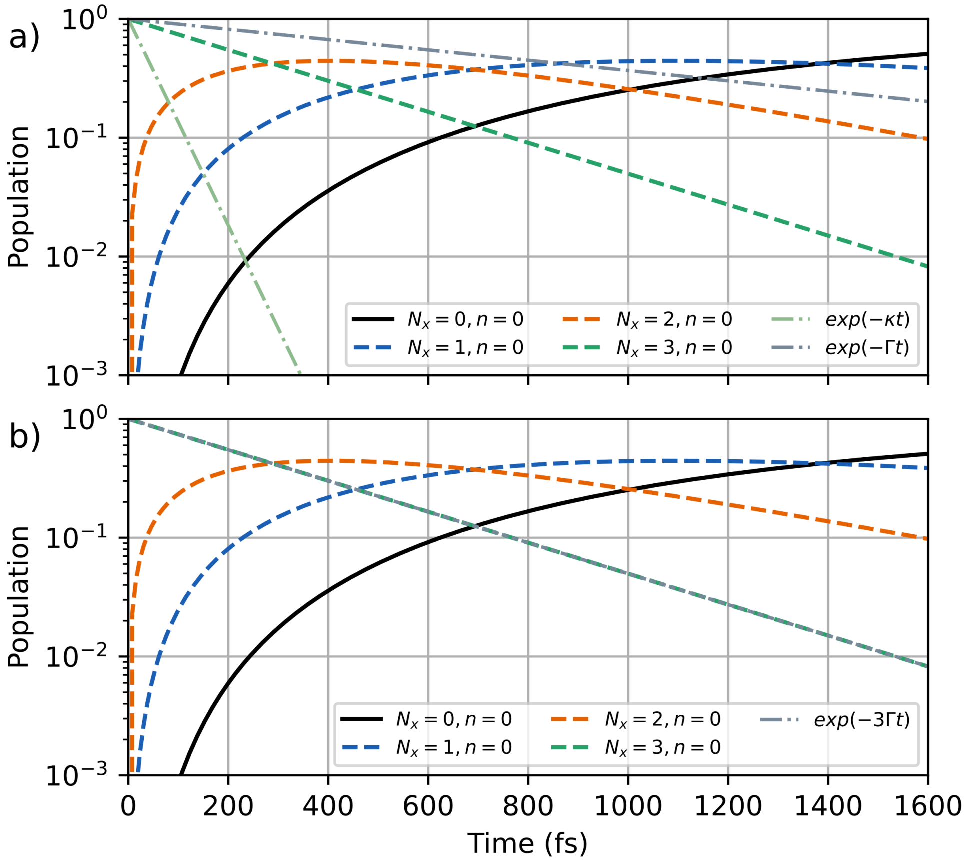

FIG. S5. Dynamics of a two-level system with $N = 8$ and an initial state that represents a (a) pure and (b) mixed state with three excitations in $|e\rangle$, without cavity coupling between levels e and g ($g_c = 0\,\mathrm{eV}$). The population is grouped by the excitation number. All additional parameters are shown in Table S1.

text.

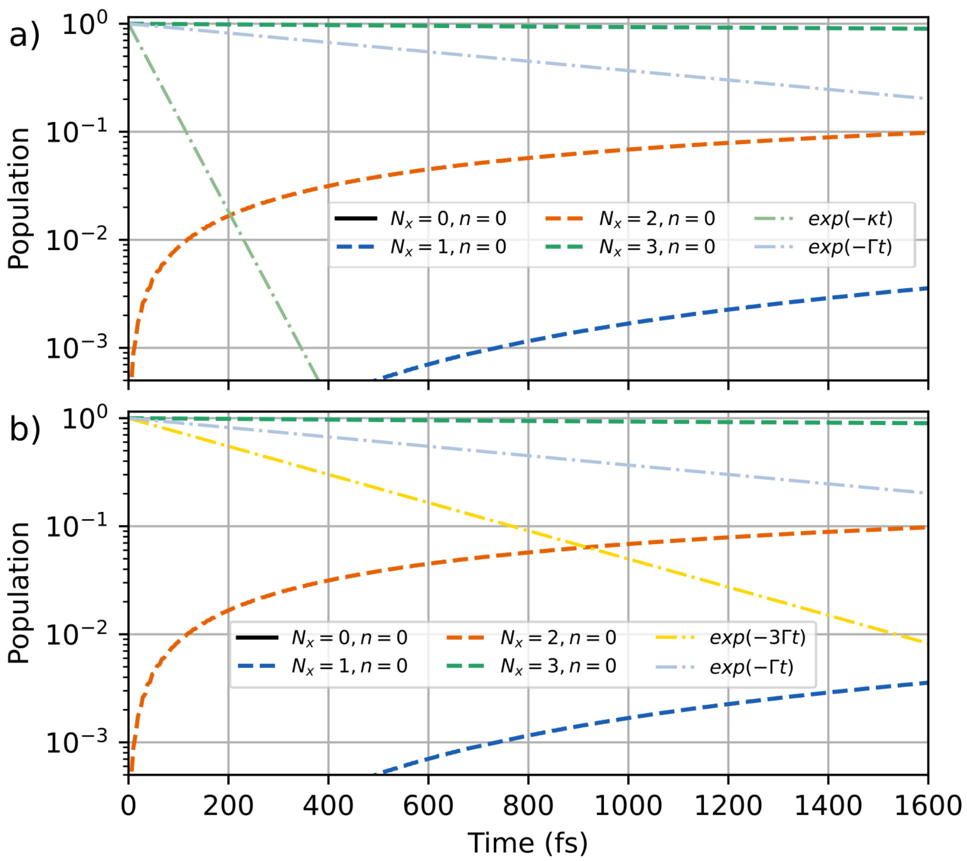

FIG. S6. Dynamics of a three-level system with $N = 8$ and an initial state that represents a (a) pure and (b) mixed state with three excitations in $|t\rangle$, without cavity coupling between levels e and g ($g_c = 0\,\mathrm{eV}$). The population is grouped by the excitation number. All additional parameters are shown in Table S1.

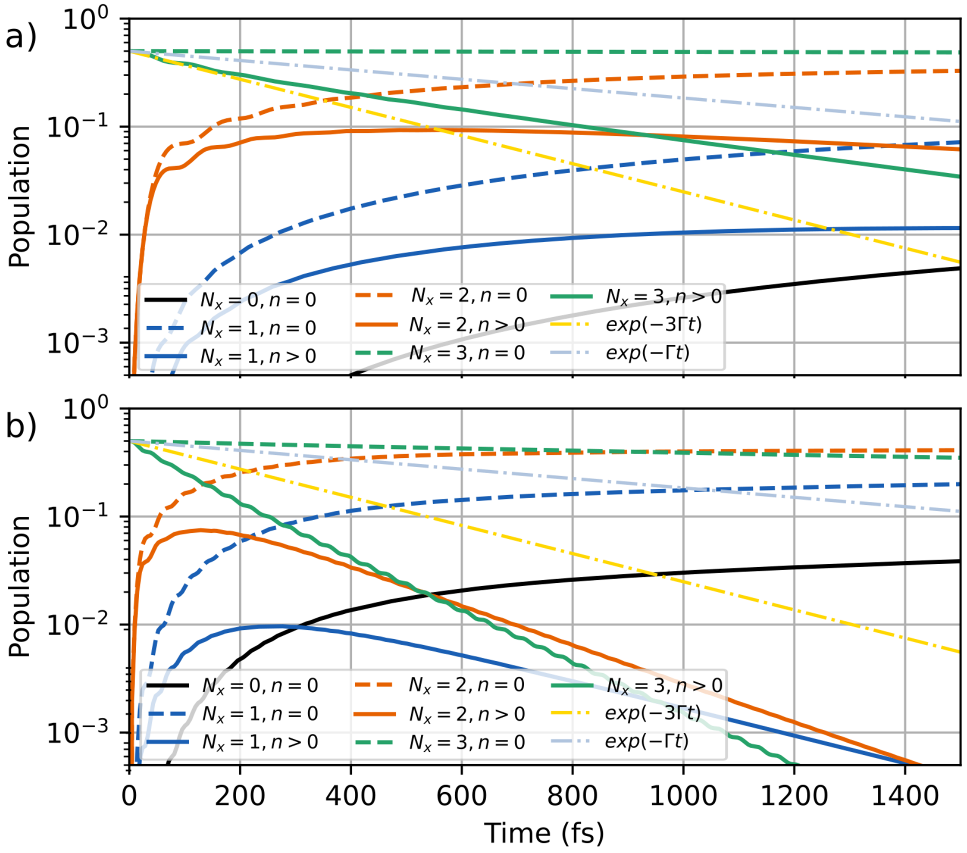

FIG. S7. Dynamics of a three-level system with $N = 8$ and an initial state that represents a mixed state with three excitations in $|t\rangle$, with coupling between levels e and t given by (a) $c_{et} = 0.025\,\mathrm{eV}$ and (b) $c_{et} = 0.1\,\mathrm{eV}$. The population is grouped by the excitation number and photonic character. All additional parameters are shown in Table S1.

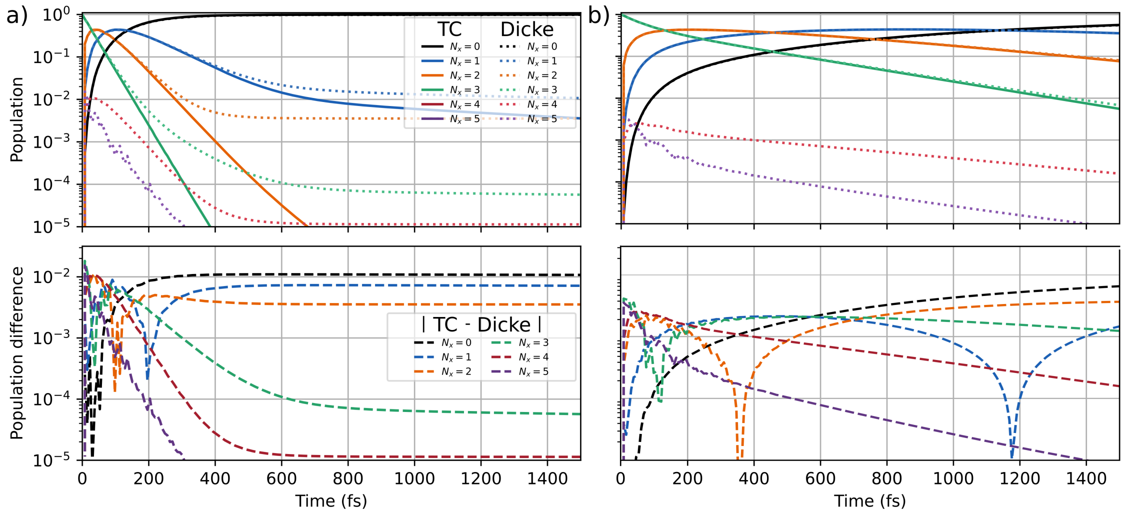

FIG. S8. Comparison of the open system dynamics between the Tavis-Cummings model (with rotating wave approximation) and the Dicke model (including counter-rotating terms). The system is comprised of $N = 8$ two-level emitters coupled to a cavity mode, initially in a pure (a) and in a mixed (b) states with $N_x = 3$. Parameters: $g_c^N = 0.5\text{eV}, \omega_c = \omega_{eg} = 4.3\text{eV}, \kappa = 1/50\text{fs}, \Gamma = 1/1000\text{fs}$.

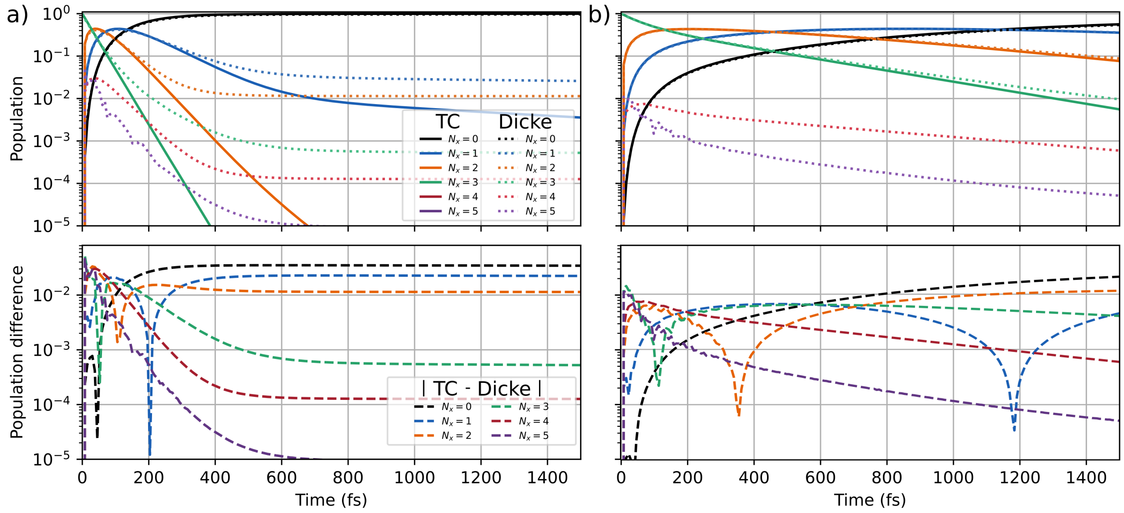

FIG. S9. Comparison of the open system dynamics between the Tavis-Cummings model (with rotating wave approximation) and the Dicke model (including counter-rotating terms). The system is comprised of $N = 8$ two-level emitters coupled to a cavity mode, initially in a pure (a) and in a mixed (b) states with $N_x = 3$. Parameters: $g_c^N = 0.86\text{eV}, \omega_c = \omega_{eg} = 4.3\text{eV}, \kappa = 1/50\text{fs}, \Gamma = 1/1000\text{fs}$.

[1] S. Andrilli and D. Hecker, Systems of linear equations, in *Elementary Linear Algebra*, edited by S. Andrilli and D. Hecker (Elsevier, 2010) pp. 79–142, sixth edition ed.

[2] R. E. Cline and R. J. Plemmons, Siam Review **18**, 92 (1976).

[3] V. Rokaj, D. M. Welakuh, M. Ruggenthaler, and A. Rubio, J. Phys. B At. Mol. Opt. Phys. **51**, 034005 (2018).

[4] C. Schäfer, M. Ruggenthaler, V. Rokaj, and A. Rubio, ACS Photonics **7**, 975 (2020).

[5] L. Borges, T. Schnappinger, and M. Kowalewski, J. Chem. Phys. **161**, 044119 (2024).
